# Special Issue “Advances in Thrombocytopenia”

**DOI:** 10.3390/jcm11226679

**Published:** 2022-11-10

**Authors:** Lili Ji, Yunfeng Cheng

**Affiliations:** 1 Department of Hematology, Zhongshan Hospital, Fudan University, Shanghai 200032, China; 2 Institute of Clinical Science, Zhongshan Hospital, Fudan University, Shanghai 200032, China; 3 Department of Hematology, Zhongshan Hospital Qingpu Branch, Fudan University, Shanghai 201700, China; 4 Center for Tumor Diagnosis & Therapy, Jinshan Hospital, Fudan University, Shanghai 201508, China

Thrombocytopenia is a commonly encountered hematologic challenge in medicine. Major types of thrombocytopenia include primary immune thrombocytopenia, drug-induced thrombocytopenia, heparin-induced thrombocytopenia, thrombotic thrombocytopenia, disseminated intravascular coagulation, gestational thrombocytopenia, and inherited thrombocytopenia. Secondary thrombocytopenia is caused by various entities such as infectious diseases, alloimmune, inhibition of bone marrow hematopoiesis, decreased megakaryocyte generation for physical and chemical factors, and benign and malignant diseases of the hematopoietic system. Attention should be paid to thrombocytopenia secondary to systemic lupus erythematosus (SLE) and antiphospholipid syndrome. Abnormal platelet distribution such as splenomegaly and pseudothrombocytopenia should also be noted. 

The current Special Issue, “Advances in Thrombocytopenia”, in the *Journal of Clinical Medicine*, is dedicated to collecting high-quality scientific contributions that mainly focus on thrombocytopenia.

Platelet premature destruction is often caused by the immune process. Primary immune thrombocytopenia (ITP) is the most common cause of thrombocytopenia in hematological diseases, in which multiple immune cells play roles in attacking platelets in peripheral blood and megakaryocytes in bone marrow. This disease is a good study entity for understanding immune-mediated thrombocytopenia. Auto-antibodies against platelet glycoprotein (GP) was first described in ITP. The GPIIbIIIa antibodies conjugated with platelets and mediated phagocytose by macrophages. Another kind of GP antibody, anti-GPIbα, leads to platelet clearance in the liver via hepatocyte Ashwell–Morell receptors [1]. The auto-antibodies also attack the megakaryocytes in bone marrow, leading to decreased production of platelets [2]. Imbalance of T helper cells (Th), including Th1/Th2 and T regulatory (Treg)/Th17 imbalance, created an inflammation cytokines environment, helping B cells to produce auto-antibodies. The characteristic Th1 family cytokines profile even had diagnostic significance in ITP patients [3]. Multiple studies confirmed Treg cells’ numerical and functional abnormality in the pathogenesis in ITP [2]. Detailed Treg cell defects, including insufficient secretion of IL-10, down-expression of CD39, and suspected abnormal PD-1/PD-L1 and CTLA-4 signal pathway, were recently disclosed in ITP [4,5]. Treg cells’ subtype change could also predict the prognosis of ITP [6]. Metabolic reprogramming in T cells, a somewhat novel concept in immune disorders, was also reported in ITP [7]. This topic is a prospective direction in ITP studies. In the upstream of T cells, the macrophage subtypes imbalance and monocyte abnormality change have been proven in ITP, which could influence the imbalance of T cells [8,9,10]. Understanding the disease pathogenesis could also help us to find new treatments [4]. Thrombopoietin receptor antagonist was recommended as a preferred second line treatment because they improved refractory ITP patients’ outcome [11,12]. Other new drugs and new combination regimen, such as ATRA combined with Rituximab [13] and low-dose decitabine [14], showed encouraging results in refractory ITP.

Drug-induced thrombocytopenia (DITP) is caused by drug-dependent, platelet-reactive antibodies (DDAbs). The onset of thrombocytopenia usually occurs about 1 week after beginning the drug. The clinical relevance of drug intake and thrombocytopenia, as well as identifying the DDAbs, infers to the diagnosis of DITP. The outcome of DITP was often favorable if the criminal drug was withdrawn in time. Some old drugs were recently found to cause thrombocytopenia [15]. Moreover, many new drugs have immerged in last decade, with the prevalence of DITP noticeably increased. It seems important to keep attention on this issue and to update the list of DITP [15,16]. As checkpoint inhibitors (ICIs) are widely used in treating malignancies, thrombocytopenia caused by ICIs was reported at a 2.84% prevalence. The rescue method included steroids, IVIG, Rituximab, and TPO-RA [17]. Chemotherapy-induced thrombocytopenia is thought to be related to reversible lower megakaryopoiesis.

Heparin-induced thrombocytopenia (HIT) occurs in 0.2%–3% of patients treated with unfractionated or low-molecular-weight heparin. It is a special kind of DITP with a high thrombosis risk. A clinical scoring system, such as 4T, could attain a risk score of HIT that helps early diagnosis. Yet, to verify the existence of the PF4 antibody is still a gold standard. New approaches were used to obtain rapid confirmation [18,19]. The procoagulant platelet assay, with 98% accuracy in identifying clinically verified HIT, could also predict a higher thrombotic risk [19]. Gene prediction in HIT was proposed recently, which might be an interesting topic [20].

Thrombotic thrombocytopenia (TTP) is one kind of von Willebrand factor (vWF)-driven thrombotic disease. ADAMTS13 is a plasma metalloprotease that cleaves vWF. Congenital or acquired ADAMTS13 deficiency causes a cumulative rise in vWF, leading to micro-thrombus and thrombocytopenia. Congenital ADAMTS13 deficiency could be compensated by plasma infusion. Acquired ADAMTS13 deficiency is mostly caused by autoantibodies against ADAMTS13. Treatments targeting antibody-mediated immune disease, including plasma exchange, glucocorticoids, and Rituximab, could relieve the disease. Caplacizumab, a monoclonal antibody to von Willebrand factor (vWF), is widely used to treat TTP. Microlyse was a fusion protein targeting VWF and uPA in the meantime. Microlyse could trigger the targeted destruction of platelet–VWF complexes by localizing plasminogen activation on microthrombi. The treatment potential of this new agent was proven in animal models [21]. In the era of COVID 19, thrombocytopenia was repeatedly reported after vaccination of adenovirus-based vaccines against SARS-CoV-2 [20,22]. Vaccine-induced thrombotic thrombocytopenia (VITT), a rare and more severe condition, is a new member of TTP.

Disseminated intravascular coagulation (DIC) is a severe condition with high mortality. The current diagnosis criteria are based on overt consumptive coagulopathies, which resemble a relatively late stage of the disease. There is a need for new diagnosis criteria to confirm DIC at an earlier stage to initiate earlier treatment. More effective treatment is also anticipated to improve the outcome. So, it is important to deepen the understanding of DIC pathophysiology. DIC involves overlapping host defense pathways, such as the uncontrolled activation of coagulation, platelets, fibrinolysis, complement, innate immunity, and inflammation, within a dysfunctional microcirculation, characterized by widespread endotheliopathy.

Gestation is a special physiologic situation. Hemolysis, elevated liver enzymes, and low platelets (HELLP) syndrome is a serious lethal disease. It appears as severe thrombocytopenia and microangiopathic hemolytic anemia, which needs more attention regarding both mother and fetus [23]. Immune thrombocytopenia could change during pregnancy and the treatment should be adjusted. ITP could recur or exacerbate during this period. Postpartum hemorrhage is another big concern in gestational thrombocytopenia. Efforts were made to predict the possibility of severe postpartum hemorrhage. Platelet count was a direct effector, while plateletcrit and platelet distribution width helped the forecast [24]. A MONITOR model could predict the postpartum hemorrhage in ITP pregnancies [25].

Inherited thrombocytopenia is composed of a pile of diseases. Gene mutations related to platelet GPs, megakaryocytes, vWF, or ADAMTS13 can all lead to thrombocytopenia [26,27,28,29,30,31] and bleeding of different severities [32]. Next-generation sequencing helps to detect the mutations. Some congenital thrombocytopenia was even found to be related to myeloid neoplasms [33] or lymphoproliferative disease on clonal evolution [26]. Congenital TTP (cTTP) is a rare hereditary disease with decreased activity of ADAMTS13. Pregnancy is identified as a trigger for TTP episodes in patients with cTTP [29]. Prophylaxis regular plasma infusion provides the conventional therapy [34], which should increase the dosage to more than 5 mL/kg/wk at the beginning of pregnancy [29]. For severe episodes of cTTP, caplacizumab was reported to be effective [35]. Gene therapy seems promising for cTTP [29] and other congenital thrombocytopenia. Some congenital amegakaryocytic thrombocytopenia could be rescued by eltrombopag [28].

Severe thrombocytopenia is often thought to be accompanied with a high risk of bleeding. On the other hand, thrombosis occurs in thrombocytopenia patients, such as HIT, TTP, ITP, and advanced liver diseases, andcritically ill patients with sepsis. Further insights showed that hemostasis depended on multiple factors. In the real world, many thrombocytopenia patients need anticoagulation for comorbidity. How can we balance the risk of thrombosis and bleeding? Few studies deal with the dilemma. TH2 (Thrombosis and Thrombocytopenia 2) score was a primitive formula that helped the decision [36]. Based on the hemostasis factors mentioned above, more powerful and more specific rules need to be figure out [37].

According to the above-mentioned topics, in this Special Issue, studies of primary and secondary thrombocytopenia are welcomed. Additionally, this Special Issue will collect excellent clinical and basic research work in this field.

As the Guest Editors, we would like to sincerely thank and appreciate the reviewers for their insightful remarks and the *JCM* team’s support. Finally, we thank all of the contributing authors for their valuable input.

## References

[1] Li J., van der Wal D.E., Zhu G., Xu M., Yougbare I., Ma L., Vadasz B., Carrim N., Grozovsky R., Ruan M. (2015). Desialylation is a mechanism of Fc-independent platelet clearance and a therapeutic target in immune thrombocytopenia. Nat. Commun..

[2] Cooper N., Solomon C.G., Ghanima W. (2019). Immune Thrombocytopenia. N. Engl. J. Med..

[3] Liu Y., Hua F., Zhan Y., Yang Y., Xie J., Cheng Y., Li F. (2021). Carcinoma associated fibroblasts small extracellular vesicles with low miR-7641 promotes breast cancer stemness and glycolysis by HIF-1α. Cell Death Discov..

[4] Ji L., Cheng Y. (2022). Treg cell abnormality and its potential treatments in patients with primary immune thrombocytopenia. Clin. Transl. Discov..

[5] Lu Y., Cheng L., Li F., Ji L., Shao X., Wu B., Zhan Y., Liu C., Min Z., Ke Y. (2019). The abnormal function of CD39(+) regulatory T cells could be corrected by high-dose dexamethasone in patients with primary immune thrombocytopenia. Ann. Hematol..

[6] Cheng L., Liu C., Li F., Wu B., Min Z., Chen P., Zhan Y., Ke Y., Hua F., Yuan L. (2019). The prediction value of Treg cell subtype alterations for glucocorticoid treatment in newly diagnosed primary immune thrombocytopenia patients. Thromb. Res..

[7] Qian M., Zhu B., Zhan Y., Wang L., Shen Q., Zhang M., Yue L., Wu D., Chen H., Wang X. (2022). Analysis of Negative Results of Metagenomics Next-Generation Sequencing in Clinical Practice. Front. Cell. Infect. Microbiol..

[8] Feng Q., Xu M., Yu Y.Y., Hou Y., Mi X., Sun Y.X., Ma S., Zuo X.Y., Shao L.L., Hou M. (2017). High-dose dexamethasone or all-trans-retinoic acid restores the balance of macrophages towards M2 in immune thrombocytopenia. J. Thromb. Haemost. JTH.

[9] Liu X.G., Liu S., Feng Q., Liu X.N., Li G.S., Sheng Z., Chen P., Liu Y., Wei Y., Dong X.Y. (2016). Thrombopoietin receptor agonists shift the balance of Fcγ receptors toward inhibitory receptor IIb on monocytes in ITP. Blood.

[10] Zhao Y., Xu P., Guo L., Wang H., Min Y., Feng Q., Hou Y., Sun T., Li G., Ji X. (2021). Tumor Necrosis Factor-α Blockade Corrects Monocyte/Macrophage Imbalance in Primary Immune Thrombocytopenia. Thromb. Haemost..

[11] Neunert C., Terrell D.R., Arnold D.M., Buchanan G., Cines D.B., Cooper N., Cuker A., Despotovic J.M., George J.N., Grace R.F. (2019). American Society of Hematology 2019 guidelines for immune thrombocytopenia. Blood Adv..

[12] Provan D., Arnold D.M., Bussel J.B., Chong B.H., Cooper N., Gernsheimer T., Ghanima W., Godeau B., González-López T.J., Grainger J. (2019). Updated international consensus report on the investigation and management of primary immune thrombocytopenia. Blood Adv..

[13] Wu Y.J., Liu H., Zeng Q.Z., Liu Y., Wang J.W., Wang W.S., Jia F., Zhou H.B., Huang Q.S., He Y. (2022). All-trans retinoic acid plus low-dose rituximab vs low-dose rituximab in corticosteroid-resistant or relapsed ITP. Blood.

[14] Zhou H., Qin P., Liu Q., Yuan C., Hao Y., Zhang H., Wang Z., Ran X., Chu X., Yu W. (2019). A prospective, multicenter study of low dose decitabine in adult patients with refractory immune thrombocytopenia. Am. J. Hematol..

[15] Nusrat S., Borogovac A., George J.N., Curtis B.R., Reese J.A. (2022). Drug (vaccine)-induced thrombocytopenia 2021: Diversity of pathogenesis and clinical features. Am. J. Hematol..

[16] Fuentes S., Chrétien B., Dolladille C., Alexandre J., Dumont A., Nguyen A., de Boysson H., Chèze S., Maigné G., Aouba A. (2022). An updated list of drugs suspected to be associated with immune thrombocytopenia based on the WHO pharmacovigilance database. Blood.

[17] Ghanem P., Marrone K., Shanbhag S., Brahmer J.R., Naik R.P. (2022). Current challenges of hematologic complications due to immune checkpoint blockade: A comprehensive review. Ann. Hematol..

[18] Rittener-Ruff L., Marchetti M., Matthey-Guirao E., Grandoni F., Gomez F.J., Alberio L. (2022). Combinations of rapid immunoassays for a speedy diagnosis of heparin-induced thrombocytopenia. J. Thromb. Haemost. JTH.

[19] Lee C.S.M., Selvadurai M.V., Pasalic L., Yeung J., Konda M., Kershaw G.W., Favaloro E.J., Chen V.M. (2022). Measurement of procoagulant platelets provides mechanistic insight and diagnostic potential in heparin-induced thrombocytopenia. J. Thromb. Haemost. JTH.

[20] Favaloro E.J., Clifford J., Leitinger E., Parker M., Sung P., Chunilal S., Tran H., Kershaw G., Fu S., Passam F. (2022). Assessment of immunological anti-platelet factor 4 antibodies for vaccine-induced thrombotic thrombocytopenia (VITT) in a large Australian cohort: A multicenter study comprising 1284 patients. J. Thromb. Haemost. JTH.

[21] de Maat S., Clark C.C., Barendrecht A.D., Smits S., van Kleef N.D., El Otmani H., Waning M., van Moorsel M., Szardenings M., Delaroque N. (2022). Microlyse: A thrombolytic agent that targets VWF for clearance of microvascular thrombosis. Blood.

[22] Nicolai L., Leunig A., Pekayvaz K., Esefeld M., Anjum A., Rath J., Riedlinger E., Ehreiser V., Mader M., Eivers L. (2022). Thrombocytopenia and splenic platelet-directed immune responses after IV ChAdOx1 nCov-19 administration. Blood.

[23] Perez Botero J., Reese J.A., George J.N., McIntosh J.J. (2021). Severe thrombocytopenia and microangiopathic hemolytic anemia in pregnancy: A guide for the consulting hematologist. Am. J. Hematol..

[24] Van Dijk W.E.M., Nijdam J.S., Haitjema S., de Groot M.C.H., Huisman A., Punt M.C., Evers A.C.C., Schutgens R.E.G., Lely A.T., van Galen K.P.M. (2021). Platelet count and indices as postpartum hemorrhage risk factors: A retrospective cohort study. J. Thromb. Haemost. JTH.

[25] Huang Q.S., Zhu X.L., Qu Q.Y., Liu X., Zhang G.C., Su Y., Chen Q., Liu F.Q., Sun X.Y., Liang M.Y. (2021). Prediction of postpartum hemorrhage in pregnant women with immune thrombocytopenia: Development and validation of the MONITOR model in a nationwide multicenter study. Am. J. Hematol..

[26] Martin E.S., Ferrer A., Mangaonkar A.A., Khan S.P., Kohorst M.A., Joshi A.Y., Hogan W.J., Olteanu H., Moyer A.M., Al-Kali A. (2021). Spectrum of hematological malignancies, clonal evolution and outcomes in 144 Mayo Clinic patients with germline predisposition syndromes. Am. J. Hematol..

[27] Scully M. (2021). Congenital TTP: Next stop, acuity and therapy. Blood.

[28] Basso-Valentina F., Levy G., Varghese L.N., Oufadem M., Neven B., Boussard C., Balayn N., Marty C., Vainchenker W., Plo I. (2021). CALR mutant protein rescues the response of MPL p.R464G variant associated with CAMT to eltrombopag. Blood.

[29] Sakai K., Fujimura Y., Nagata Y., Higasa S., Moriyama M., Isonishi A., Konno M., Kajiwara M., Ogawa Y., Kaburagi S. (2020). Success and limitations of plasma treatment in pregnant women with congenital thrombotic thrombocytopenic purpura. J. Thromb. Haemost. JTH.

[30] Quach M.E., Li R. (2020). Structure-function of platelet glycoprotein Ib-IX. J. Thromb. Haemost. JTH.

[31] Pal K., Nowak R., Billington N., Liu R., Ghosh A., Sellers J.R., Fowler V.M. (2020). Megakaryocyte migration defects due to nonmuscle myosin IIA mutations underlie thrombocytopenia in MYH9-related disease. Blood.

[32] Blaauwgeers M.W., Kruip M., Beckers E.A.M., Coppens M., Eikenboom J., van Galen K.P.M., Tamminga R.Y.J., Urbanus R.T., Schutgens R.E.G. (2020). Bleeding phenotype and diagnostic characterization of patients with congenital platelet defects. Am. J. Hematol..

[33] Sakurai M., Nannya Y., Yamazaki R., Yamaguchi K., Koda Y., Abe R., Yokoyama K., Ogawa S., Mori T. (2022). Germline RUNX1 translocation in familial platelet disorder with propensity to myeloid malignancies. Ann. Hematol..

[34] Tarasco E., Bütikofer L., Friedman K.D., George J.N., Hrachovinova I., Knöbl P.N., Matsumoto M., von Krogh A.S., Aebi-Huber I., Cermakova Z. (2021). Annual incidence and severity of acute episodes in hereditary thrombotic thrombocytopenic purpura. Blood.

[35] Boothby A., Mazepa M. (2022). Caplacizumab for congenital thrombotic thrombocytopenic purpura. Am. J. Hematol..

[36] Balitsky A.K., Kelton J.G., Arnold D.M. (2018). Managing antithrombotic therapy in immune thrombocytopenia: Development of the TH2 risk assessment score. Blood.

[37] Thachil J., Carrier M., Lisman T. (2022). Anticoagulation in thrombocytopenic patients—Time to rethink?. J. Thromb. Haemost. JTH.

